# Simultaneous cardio-cerebral infarction in the coronavirus disease pandemic era: A case series

**DOI:** 10.1097/MD.0000000000024496

**Published:** 2021-01-29

**Authors:** Rawan Eskandarani, Seham Sahli, Shaima Sawan, Asim Alsaeed

**Affiliations:** Emergency Medicine Administration, King Fahad Medical City, Riyadh, Kingdom of Saudi Arabia.

**Keywords:** coronavirus disease, infarction, ischemic, myocardial, stroke

## Abstract

**Introduction:**

Simultaneous occurrence of acute ischemic stroke and myocardial infarction is reported to have variable precipitating causes. This occurrence has been rarely reported in the literature and described only in very few case reports. During the surge of coronavirus disease (COVID-19) in our region, we noted an increase in the simultaneous occurrence of cardio-cerebral infarction. This led us to explore the possible mechanisms and pathophysiology that could contribute to this increase. The retrospective nature of the study limited us from drawing any conclusion about causation. Rather, we aimed to formulate a hypothesis for future, more rigorous studies.

**Patient concerns:**

We present an overview of 5 cases of simultaneous cardio-cerebral infarction that we encountered in our emergency department within 1 month.

**Diagnosis:**

In all cases, diagnosis was confirmed using an electrocardiogram, assessment of laboratory cardiac markers, and imaging.

**Interventions:**

In all cases, dual antiplatelet therapy was started and thrombolysis was held, as the condition was considered high risk in most of the patients. Cardiac catheterization lab was not activated either because the patient was unstable or the risk of COVID-19 in staff outweighed the benefit added in patient treatment.

**Outcomes:**

Two out of 5 patients died because of early complications that lasted for few days. The remaining 3 were discharged from the hospital in moderate functionality for extensive therapy and rehabilitation.

**Conclusion:**

Early recognition and immediate treatment is important in different scenarios leading to thrombosis as the outcome. Additionally, addressing the unknown risks that could contribute to our traditional understanding of these causative mechanisms is important. The hypothesis of exacerbated damage caused by inflammatory and immunological endothelial systemic damage should further be explored to be able to delineate new possibilities in managing these conditions.

## 1 Introduction

**Figure 1:**
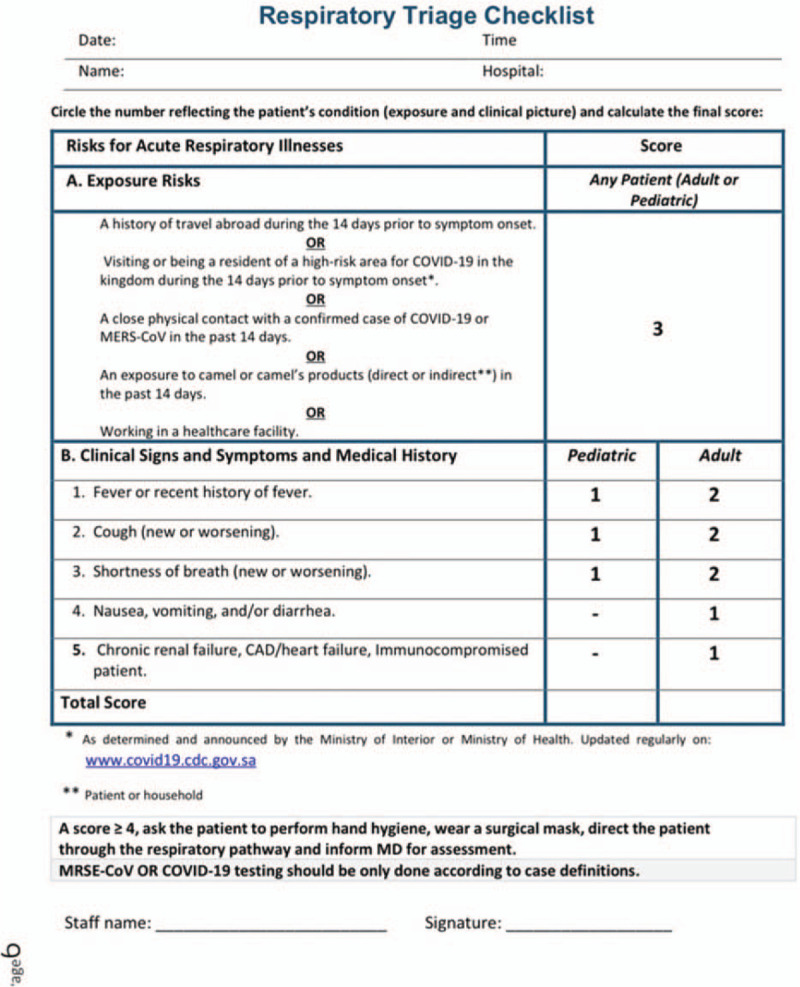
Triage checklist used to determine the risk of exposure.

The simultaneous occurrence of acute ischemic stroke and myocardial infarction is not very common. This occurrence has been reported in the literature with variable precipitating causes.^[1–4]^ In the Austrian stroke unit registry, the incidence of myocardial infarction among patients with transient ischemic attack or ischemic stroke during treatment in the stroke unit was found to be 1%.^[5]^ During the coronavirus disease (COVID-19) pandemic due to severe acute respiratory syndrome coronavirus 2 (SARS-CoV-2) infection, a stronger association between COVID-19 and venous and large vessel arterial thromboses has been noted, with up to 25% in hospitalized patients.^[6–9]^ This association is not clearly defined, and further studies are needed to determine whether this relationship exists. During the COVID-19 pandemic, in our region, we noted an increase in cases with rare, simultaneous cardio-cerebral events within a few weeks. While we did not confirm the direct association with COVID-19 in these cases, we hypothesize that COVID-19 increased the risk of this presentation. We herein discuss in detail the possible mechanisms and pathophysiology of thrombosis that can explain this increase in the occurrence. Due to the retrospective study design, which is a limitation of this case series, we hypothesize that only COVID-19 could be a major contributing factor for the increased occurrence. Any direct association remains yet open for further studies. All cases presented here had a high score suspicion for COVID-19 based on our national COVID-19 triage criteria (Fig. 1). Four of 5 patients tested negative for COVID-19 by polymerase chain reaction (PCR) on their first sample. No repeat testing was performed to confirm the results due to different limitations. The option of immunoglobulin serology was unavailable at our institution at the time of writing this manuscript. PCR testing from the nasal swab has a 63% sensitivity and the results should be carefully interpreted with clinical and radiological presentation along with the viral detection.^[10]^ The likelihood of COVID-19 in all cases was based on the high triage score, clinical presentation, or history of contact with a COVID-19-positive patient.

## 2 Patient information

### 2.1 Case number 1

**Figure 2:**
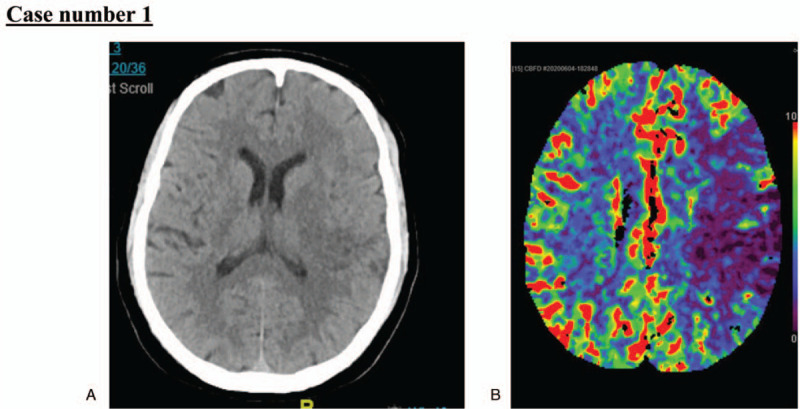
A. Unenhanced computed tomography and B. Brain perfusion scan revealing left middle cerebral artery (MCA) ischemic stroke.

**Figure 3:**
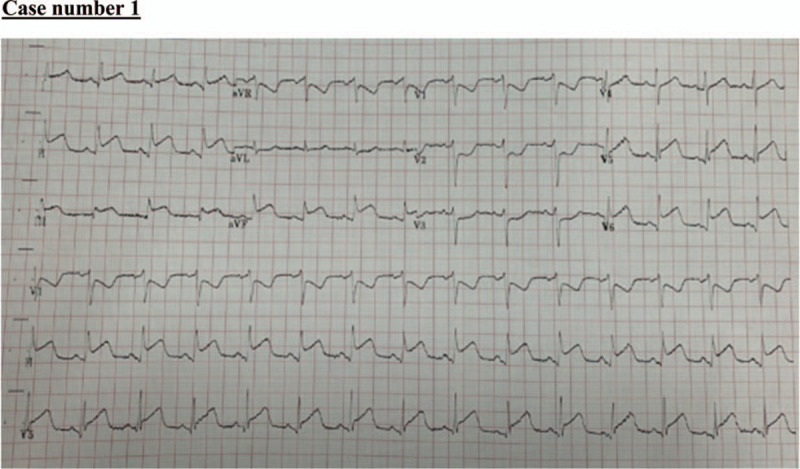
Electrocardiogram showing ST- elevations consistent with inferolateral myocardial infarction.

A 62-year-old man with a history of diabetes mellitus presented to the emergency department with sudden-onset, right-sided body weakness, and aphasia with no clear onset. According to the family, he complained of only fever and dry cough at home 3 days before presentation but denied any chest pain or other respiratory symptoms. At presentation, his initial blood pressure (BP), heart rate (HR), respiratory rate (RR), oxygen (O_2_) saturation, body temperature, and blood glucose level were 141/83 mmHg; 119 beats per minute; 48 breaths per minute; 76% at room air; 37 °C; and 252 mg/dL, respectively. He was immediately placed on 10 L oxygen which improved his saturation to 95%. At neurological examination, he was confused and uncooperative. He showed global aphasia, right sided weakness, right sided facial palsy, and left sided gaze. His National Institute of Health Stroke Scale (NIHSS) score was 22. An immediate stroke code activation was initiated as per our institutional protocol, and an initial unenhanced cranial computed tomography (CT) revealed left middle cerebral artery (MCA) ischemic stroke (Fig. 2). CT angiography showed left common carotid artery thrombosis, which suggested the possible embolic nature of his stroke. He was out of the window period, and the risk outweighed the benefit; thus, he was not considered a candidate for thrombolytic therapy. His chest x-ray revealed bilateral infiltrations that were suggestive of COVID-19. An electrocardiogram was also obtained and showed an ST-elevation consistent with inferolateral myocardial infarction (Fig. 3). His echocardiography showed inferior wall motion abnormality, and no evidence of any intracardiac thrombus or aortic dissection. An immediate multidisciplinary discussion occurred with his family to decide the best therapeutic approach to salvage the brain and myocardial tissue. Cardiac angiography option was not feasible at that time at our institution because of the COVID-19 pandemic. Thrombolytic therapy for myocardial infarction was considered to be of moderate to high risk for developing intracranial hemorrhage or alveolar hemorrhage. He was started on double antiplatelet therapy (aspirin and clopidogrel) at full loading doses, followed by 100 mg aspirin and 75 mg clopidogrel. Heparin (5000 units subcutaneous injection) was started the next day as per the institution ST-elevation myocardial infarction (STEMI) protocol. During his admission, the patient continued to undergo worsening clinically, developing severe hypoxemia, not responding to oxygen therapy, and requiring mechanical ventilation. His code status was discussed with the family, as he had a Glasgow coma scale of 3 with no sedation and no signs of brain stem reflexes. Two days after his admission, he developed asystolic cardiopulmonary arrest.

### 2.2 Case number 2

**Figure 4:**
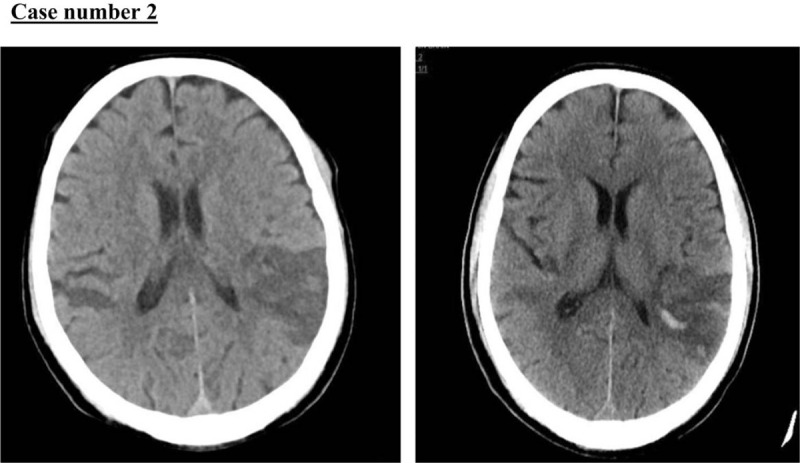
A and B unenhanced cranial computed tomography (CT) revealed multiple bilateral parietal, occipital, and cerebellar ischemic infarctions.

**Figure 5:**
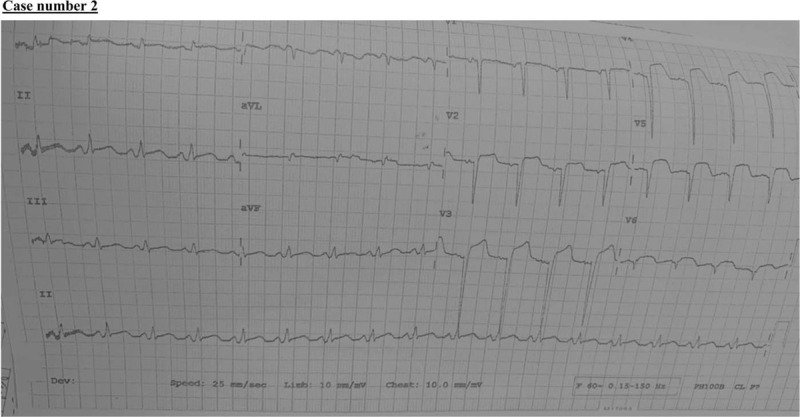
An ST-elevation consistent with antero-lateral myocardial infarction.

**Figure 6:**
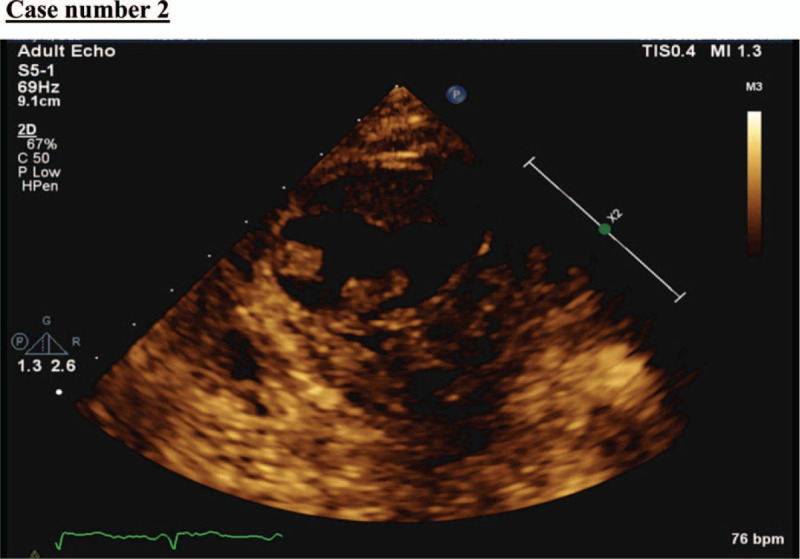
Echocardiography showed apical thrombus 5.3 × 1.7 cm in size.

A 50-year-old man, with no previous medical history, presented to the emergency department with a history of acute confusion and agitation that started the day before presentation. He had a positive history of non-documented fever at home and dry cough for 5 days before presentation. He had no associated chest pain or other respiratory symptoms. At initial assessment, his BP, HR, RR, O_2_ saturation, temperature, and blood glucose level were 127/99 mmHg; 101 beats per minute; 22 breaths per minute; 100% in room air; 36.7 °C; and 333 mg/dL, respectively. At neurological examination, he was confused, not following commands; he was aphasic, and had no facial asymmetry; and he withdrew from painful stimuli except in the right arm, which showed clear weakness. His NIHSS score was 21. The initial plain cranial CT revealed multiple bilateral parietal, occipital, and cerebellar ischemic infarctions (Fig. 4), which appeared to have an embolic origin. Electrocardiography was performed after the CT and it showed an ST-elevation consistent with antero-lateral myocardial infarction (Fig. 5). Echocardiography showed a large apical thrombus 5.3 × 1.7 cm in size (Fig. 6). The left ventricle was severely dilated with regional wall abnormalities, left ventricular wall and apical akinesia, mild inferior hypokinesia, mildly reduced right ventricular systolic function, and ejection fraction of 30%.

He also appeared to have diabetic ketoacidosis, which was managed accordingly. His chest x-ray did not show any signs of consolidation or pulmonary edema. He was started on antiplatelet and anticoagulation therapies, although administration of thrombolytics was considered high risk. Two days later, he developed intracranial hemorrhagic transformation, and anticoagulation therapy was terminated; he was maintained on a single antiplatelet therapy.

### 2.3 Case number 3

**Figure 7:**
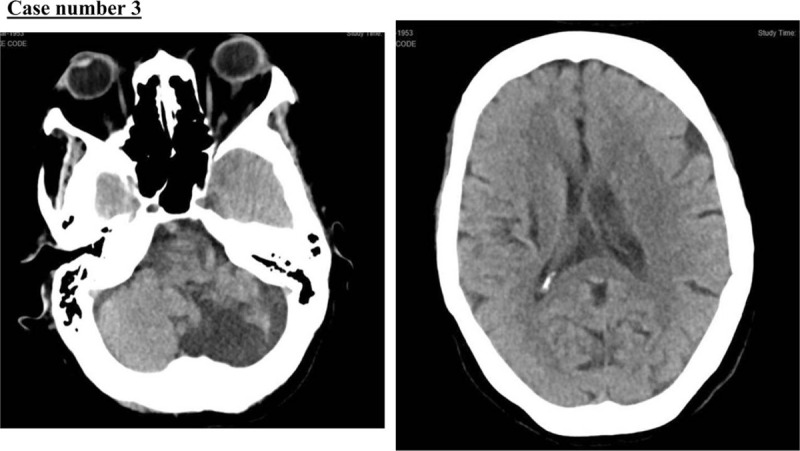
Hypodense area in the right external capsule and in the left cerebellum.

**Figure 8:**
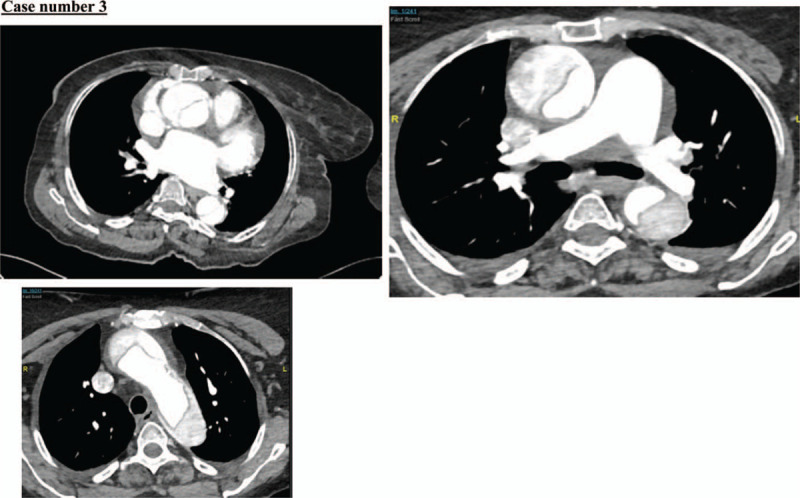
The aortic dissection extends inferiorly from the aortic root all the way to the bifurcation of the abdominal aorta.

A 67-year-old woman with a medical history of hypertension, old ischemic stroke, and atrial fibrillation, presented to our emergency department with a history of neck and chest pain followed by left-sided weakness and syncopal attack 2 hours before her presentation. Her initial vital signs, i.e., BP, HR, RR, and O_2_ saturation, were 198/181 mmHg; 70 beats per minute; 20 breaths per minute; and 98% in room air. Her NIHSS score was 14. The stroke code activation showed a hypodense area in the right external capsule and in the left cerebellum (suggestive of encephalomalacia), with both most likely related to her old ischemic insult (Fig. 7). There were no other major intracranial findings. A CT angiogram showed acute aortic dissection involving the ascending arch and descending aorta along the lateral aspect with true (3 × 2 cm) and false (4.2 × 2.5 cm) lumen, representing Stanford type A. The aortic dissection extended inferiorly from the aortic root all the way to the bifurcation of the abdominal aorta with further extension into the left common iliac, left external, and internal iliac arteries (Fig. 8).

**Figure 9:**
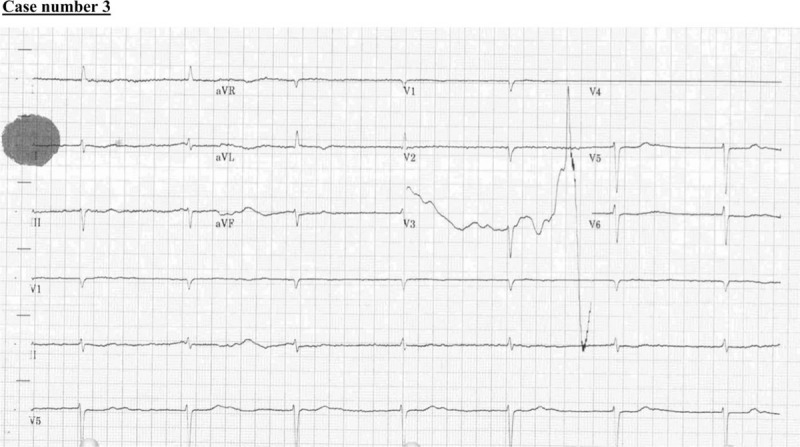
Electrocardiogram showing her bradycardia just minutes before her collapse.

There was a large filling defect seen at the base of the brachiocephalic artery representing severe right common carotid stenosis, and subsequent faint enhancement of the internal carotid, and right cerebral circulation distal branches. There was urgent consultation with the vascular surgery and cardiac surgery teams, but the patient developed cardiac arrest before going to the operation room (Fig. 9).

### 2.4 Case number 4

A 56-year-old man, not known to have any medical illness, presented to our emergency department after visiting another facility for chest pain; he was diagnosed with non-STEMI in the last 12 hours. When he presented at our institution, he had new right-sided weakness and facial deviation. His NIHSS score was 12. His initial BP, HR, RR, O_2_ saturation, and body temperature were 130/73 mmHg; 100 beats per minute; 20 breaths per minute; 93% in room air; and 37°C, respectively.

**Figure 10:**
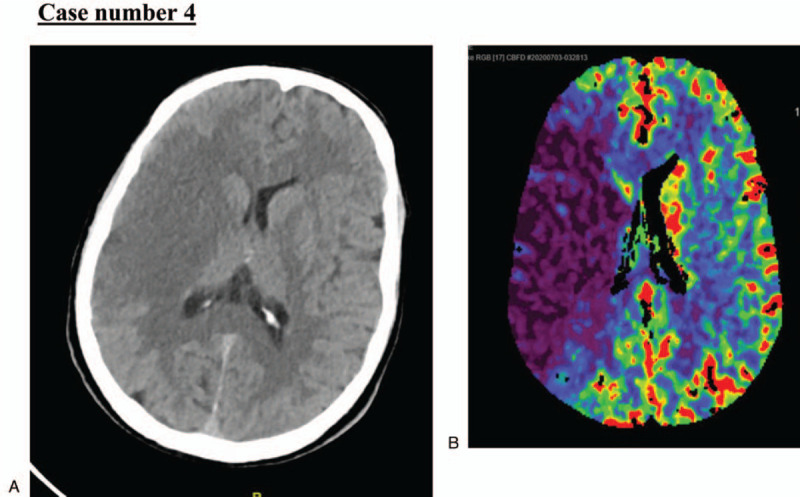
A. Plain computed tomography (CT) brain demonstrated loss of the right insular ribbon with hyperdense middle cerebral artery (MCA) sign. B. CT perfusion showed decreased cerebral flow and volume representing core infarcts with lack of areas of penumbra.

**Figure 11:**
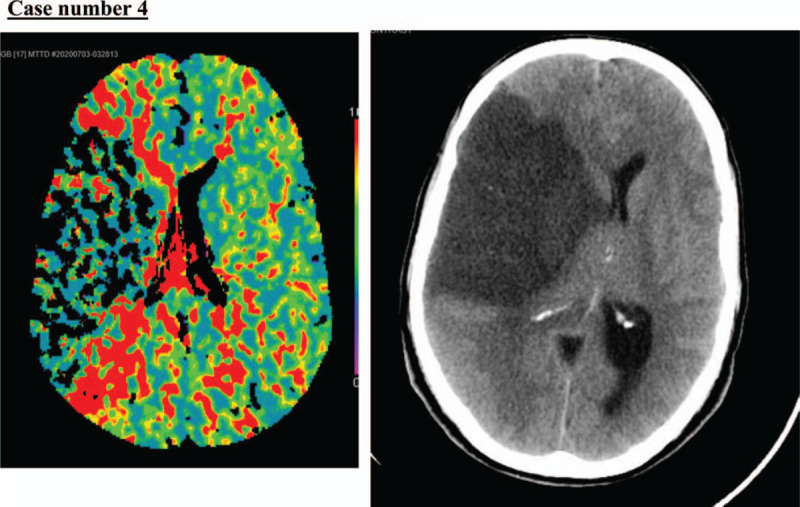
Large ischemic infarction in the right fronto-parietal-temporal lobes, causing a mass effect on the ipsilateral ventricle.

Stroke code was activated as per our institution policy. Plain CT brain demonstrated loss of the right insular ribbon with a hyperdense MCA sign (Fig. 10). Large ischemic infarction in the right fronto-parietal-temporal lobes caused a mass effect on the ipsilateral ventricle (Fig. 11). There was no evidence of a midline shift or any hemorrhagic transformation. A CT angiogram showed complete occlusion of the right internal carotid artery just after the bifurcation of the right common carotid artery, resulting in complete non-opacification of the right MCA. There was asymmetrical lack of flow within the right-sided tributaries and superficial arteries. CT perfusion showed decreased cerebral flow and volume representing core infarcts with lack of areas in the penumbra (Fig. 10). The patient's condition continued to worsen over the next few days with a decreased level of consciousness. His follow-up brain CT scan showed a progressive increase in hypodense areas with leftward midline shift of up to 13 mm and progressive mass effects. These caused subfalcine and uncal herniation; however, we noted no evidence of hemorrhagic transformation. He underwent craniotomy decompression, and his condition became reasonably stable. He was discharged home after 3-week hospital stay with decreased functionally from his admission.

### 2.5 Case number 5

**Figure 12:**
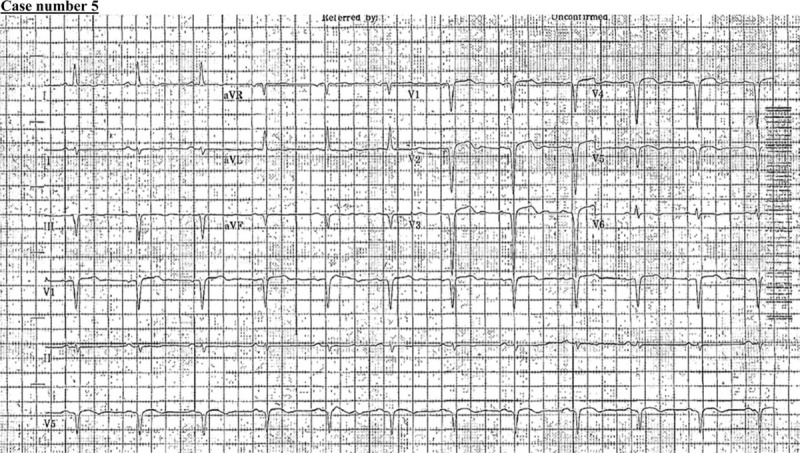
ST-elevation and anterolateral myocardial infarction.

**Figure 13:**
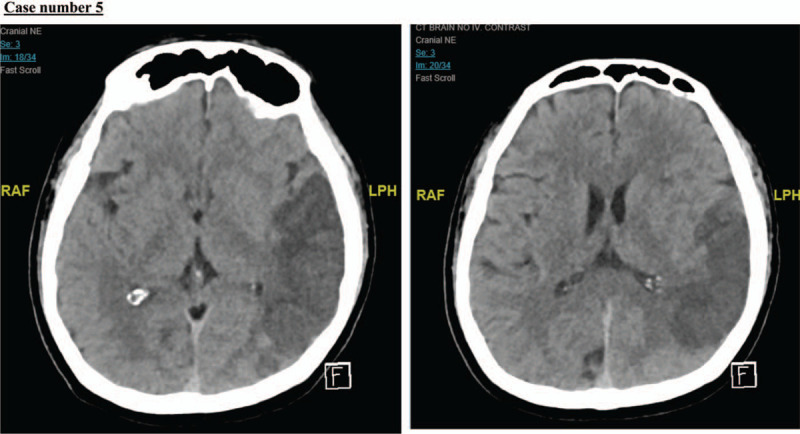
Plain computed tomography (CT) brain showed a major left middle cerebral artery (MCA), and posterior cerebral artery territory infarction with effacement of the cortical sulci.

**Figure 14:**
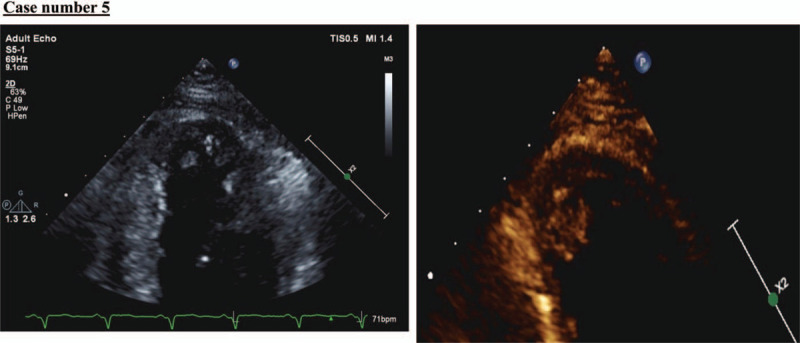
Echocardiography showing a left ventricle with a moderate sized (14 mm × 14 mm) apical thrombus.

A 50-year-old man, with a medical history of hypertension, was referred to our institution because of ST-elevation and anterolateral myocardial infarction (Fig. 12). He was in a confused state 10 hours before his presentation at our emergency department. He appeared to be confused at presentation, with no notable neurological deficit. He was alert, responding to simple commands but otherwise had difficulty in comprehension. He moved all 4 limbs against gravity with slight weakness. His plain CT brain showed a major left MCA, and posterior cerebral artery territory infarction with effacement of the cortical sulci (Fig. 13). His echocardiography showed a moderately sized left ventricle (14 mm × 14 mm) apical thrombus with an ejection fraction of 30% to 35% (Fig. 14). He remained hospitalized for 2 weeks and was discharged in good functional condition on anticoagulation therapy.

### 2.6 Ethics statement

This manuscript has been reviewed and approved by the institutional review board committee to ensure adherence and compliance as per Declaration of Helsinki principles (IRB No. 20-496).

## 3 Discussion

The simultaneous occurrence of acute ischemic stroke and myocardial infarction is rare but life-threatening. It is challenging because both conditions require timely diagnosis and management.^[11]^ The term “cardio-cerebral infarction” was coined in 2010 by Omar et al,^[12]^ and it describes the simultaneous occurrence of acute ischemic stroke and acute myocardial infarction. According to the scientific statement of the American Heart Association/American Stroke Association (AHA/ASA), it is recommended to start intravenous alteplase at a dose appropriate for cerebral ischemia, followed by percutaneous coronary angioplasty when hyperacute simultaneous cardio-cerebral infarction occurs, and this approach is considered reasonable.^[13]^ Both conditions have a high risk of mortality and a narrow therapeutic window. Delaying treatment of one, over the other, may result in irreversible damage.^[12]^ There is no evidence-based guideline for the best treatment approach or clinical studies to guide the management. The incidence is reported to be 0.009%.^[14]^ Few case reports have been described in the literature with simultaneous cardio-cerebral infarction.^[4,12,14,15]^ Multiple mechanisms can be proposed as possible causes for cardio-cerebral infarction. First, it could occur in the presence of intracardiac thrombus with poor ventricular function, which can simultaneously embolize the cerebral and coronary arteries.^[16]^ Second, arterial thromboembolism caused by atrial fibrillation is another cause for simultaneous cardio-cerebral emboli. Cardiac emboli caused by atrial fibrillation are usually large and can cause a massive infarct in the MCA.^[17]^ Third, type-I aortic dissection can cause simultaneous myocardial infarction and ischemic stroke.^[18]^ Fourth, right ventricular infarction with a patent foramen ovale can embolize to vascular territories and cause an ischemic stroke.^[12]^

This case series describes 5 cases from the ongoing COVID-19 pandemic. We suggest that COVID-19 be considered a high-risk factor for ischemic stroke, myocardial infarction, and systemic thrombosis. Vascular endothelial dysfunction and coagulopathy are the likely etiologies.^[19]^

The incidence of stroke in a retrospective study in Wuhan, China, showed an incidence of 5% among hospitalized patients infected with SARS-CoV-2.^[20]^ In a Dutch study, it was found that 31% of COVID-19 patients admitted to the intensive care unit had thrombotic complications. Of those, 3.7% were arterial thrombotic events.^[21]^ The elevated levels of D-dimer and C reactive protein indicate an increased inflammatory and an abnormal coagulopathy state. The ischemic manifestations of COVID-19 could be explained by this increase in the inflammatory state, abnormal coagulopathy, cytokine storm, viral-mediated disruption of the endothelium.^[21–23]^ These all have been hypothesized to cause an increase in the risk of large vessel arterial thrombosis in addition to the postulated increase in venous thrombosis and COVID-19-related microangiopathy.^[6]^ The pathogenic mechanism of this relationship is still to be determined in further studies.

In hospitalized patients in Wuhan, China, it was found that 36.7% of COVID-19 patients had neurological manifestations.^[24]^ It is proposed that SARS-CoV-2 enters the central nervous system (CNS) through the hematogenous or retrograde neuronal route, similar to other respiratory viruses.^[24]^ In one case, SARS-CoV-2 was identified in the cerebrospinal fluid by PCR.^[25]^ Other reports were not able to identify the virus in the cerebrospinal fluid despite positive COVID-19 PCR in nasal swabs with concurrent neurological manifestations.^[26]^

**Figure 15:**
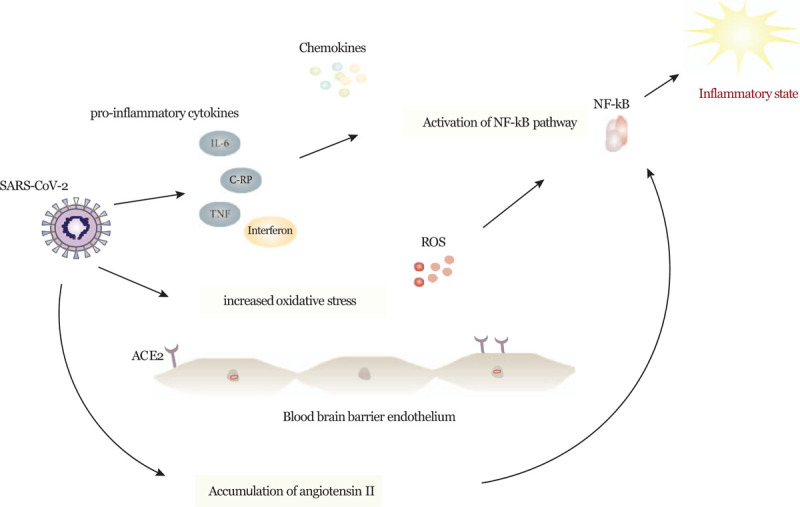
Proposed neurotropic mechanisms of the severe acute respiratory syndrome coronavirus (SARS-CoV-2). ACE2 = angiotensin-converting enzyme 2, C-RP = C-reactive protein, IL-6 = interleuking-6, NF- κB = nuclear factor kappa, ROS = reactive oxygen species, TNF = tumor necrosis factor.

The underlying neurotropic mechanisms of COVID-19 are yet to be fully understood.^[27]^ In previous SARS viruses, it has been postulated that the SARS-CoV-1 can infect and invade the blood-brain barrier (BBB) endothelial cells, allowing direct passage of the virus across the BBB into the CNS.^[28]^ Binding of the virus to the angiotensin-converting enzyme 2 (ACE2) receptors expressed in the capillary endothelium of the BBB has been proposed to have a major role in the direct viral access into the CNS.^[27,29]^ Depletion of ACE2 receptors may play a major role in endothelial damage and overexpression of angiotensin II because of the unopposed ACE1 receptors. Accumulation of angiotensin II can also activate the nuclear factor-kappa (NF-κB)-signaling pathway and the interleuking-6 (IL-6) pathway.^[30]^ The activation of the NF-κB pathway has an important role in the pathogenesis of inflammatory diseases (Fig. 15).^[31,32]^ This is accompanied by an age-related decline in the ACE2 receptors and can lead to the proinflammatory end-organ tissue damage and wide systemic endothelial dysfunction and microangiopathic abnormalities seen in COVID-19.^[33,34]^ ACE2 receptor overexpression has been established to play a protective role in ischemic stroke.^[35,36]^

Even though respiratory symptoms in COVID-19 are predominant, neurological, and cardiovascular manifestations in patients with pre-existing conditions have been noted to be potentially responsible for worse outcomes. Multiorgan damage has been noted more with increased viral replication, increased cytokine release storm, and exaggerated inflammatory and coagulopathic states.^[37,38]^

Increased pro-inflammatory cytokines (IL)-6, tumor necrosis factor (TNF)-α, interferons (IFNs), and chemokines (CXC, CC, C, and CX3C) are likely related to the extensive systemic inflammation and more severe multi-organ involvement (Table 1).^[39,40]^ Multiple risk factors have been suggested to increase the morbidity and mortality and are linked to worsening outcome and multiorgan involvement such as advanced age, prior medical illness, and obesity.^[19,41–43]^

**Table 1:** Proposed mechanisms of Covid-19 pathophysiology.

Increased pro-inflammatory cytokines
Increased Chemokines
Increase oxidative status
Activation of NF-κB pathway
Imbalance of the RAAS System

NF-κB = Nuclear Factor-Kappa, RAAS = Renin-Angiotensin-Aldosterone System.

The inflammatory damage by the renin-angiotensin-aldosterone system (RAAS) is further exacerbated by the oxidative stress caused by increased reactive oxygen species (ROS). Oxidative stress, mainly Toll-like receptor 4 pathway, is thought to be triggered by viral pathogens such as SARS-CoV-2, amplifying host inflammatory response.^[44]^ Even though the role of ROS has been implicated in different pathologies such as cancer, atherosclerosis, and neurodegenerative diseases; the brain is particularly sensitive to oxidative stress and excessive production of ROS.^[45]^ ROS imbalance is further exacerbated by the endothelial disruption and conformational changes that interrupts cell-to-cell adhesion inducing BBB breakdown, viral invasion, and inflammatory mediator infiltration.^[46]^

The oxygen-depleted ischemic cells have increased acidosis, which will further exacerbate the conversion of superoxide radical (O^2–^) into hydrogen peroxide (H_2_O_2_) and the more reactive hydroxyl radical (OH).^[47]^ The different free radical species affect many processes within the brain parenchyma and can result in detrimental cellular effects such as lipid peroxidation, affecting cellular signaling and initiating apoptosis.^[48,49]^ The activation of the NF-κB pathway have an important role in the pathogenesis of inflammatory diseases.^[31,32]^ When stimulated by the release of TNFα, or other cell stressors, the BNF-κ pathway can have a neuroprotective or a proinflammatory role; and influence cell regulation and survival based on the tissue location and pathological state.^[50]^ The binding of the COVID-19 virus to the ACE2 receptors can also activate BNF-κ.^[51]^ Angiotensin II accumulation will lead to increased vasoconstriction, increased oxidative stress, and inflammation.^[52]^

The unique features of COVID-19 pathophysiology make it more challenging to tackle and make it an open area for further studies to delineate the best modality of treatment. Immune modulatory therapy is an area for further research because it is proposed to have a major role in the COVID-19 pathophysiology. The inflammatory dysregulation is further worsened with chronic conditions that have similar immunological disruption such as diabetes mellitus and dyslipidemia. This could explain the COVID-19 exacerbation of existing chronic conditions with increasing incidence of COVID-19-related thrombotic cardio-cerebral events.^[8,53]^

The proposed systemic viral endothelial disruption, inflammatory dysregulation, cytokine storm, ROS imbalance, and RAAS disturbance can all play a major role in macro-thrombosis formation. The relation of COVID-19 with arterial thrombosis should be further explored. The treatment approaches for simultaneous STEMI and acute ischemic stroke are extremely challenging with a high risk of morbidity and mortality even in the regular circumstances and much more so in the COVID-19-related settings. In the context of simultaneous cardio-cerebral infarction, the AHA/ASA in 2018 recommended that intravenous alteplase can be used at the same dose for cerebral ischemia, followed by percutaneous coronary intervention.^[54]^ Despite this recommendation, it is challenging to treat simultaneous cardio-cerebral infarction because of different risks including time of presentation, different dosing requirements, availability of endovascular therapy, and the timing of fibrinolytic therapy required for the different conditions. Individualized treatment approaches are recommended to be considered according to the different operating factors.^[11,55]^

## 4 Conclusion

In the cases mentioned above, different treatment approaches were considered based on the individual presentation and risk of bleeding. Cardio-cerebral infarction is challenging concerning diagnosis, and treatment is not straightforward. If COVID-19 infection is to be considered, then this challenge is further complicated, as pathophysiology and treatment approaches are not yet fully determined. Despite the retrospective study design, which is a limitation in our cases, COVID-19 should always be considered and further testing strategies should be considered, if available, because the pathophysiology is different and requires different treatment modalities and strategies. To date, the testing modalities and immunological effects of COVID-19 are not fully understood. New treatment strategies such as antivirals, antioxidants, anti-inflammatory agents, targeted immunomodulatory therapies, antifibrotics, and anticoagulants could prove beneficial in these cases, and further research is warranted.

## Acknowledgments

The authors would like to acknowledge Dr. Areej Almweisheer and Dr. Fai Aldosari for their help in tracing back patients’ events upon admission.

## Author contributions

**Conceptualization:** Rawan Eskandarani, Seham Sahli.

**Data curation:** Rawan Eskandarani, Seham Sahli, Asim Alsaeed.

**Investigation:** Rawan Eskandarani, Seham Sahli, Shaima Sawan, Asim Alsaeed.

**Supervision:** Rawan Eskandarani, Asim Alsaeed.

**Writing – original draft:** Rawan Eskandarani, Seham Sahli, Shaima Sawan.

**Writing – review & editing:** Rawan Eskandarani, Asim Alsaeed.

## Abbreviations

Abbreviations: ACE = angiotensin-converting enzyme 2, AHA/ASA = American Heart Association/American Stroke Association, BBB = blood-brain barrier, CNS = central nervous system, COVID-19 = coronavirus disease, CT = computed tomography, MCA = middle cerebral artery, NF-κB = Nuclear Factor-Kappa, NIHSS = National Institute of Health Stroke Scale, PCR = polymerase chain reaction, RAAS = Renin-Angiotensin-Aldosterone System, ROS = reactive oxygen species, SARS-CoV-2 = severe acute respiratory syndrome coronavirus 2, STEMI = ST-elevation myocardial infarction, TNF = tumor necrosis factor.

## References

[1] Zaidi WW Khoo CS Remli R. A case report of acute ischaemic stroke with concurrent acute ST-elevation myocardial infarction: can you thrombolyse? J Neurol Sci 2017;381:1119.

[2] Kijpaisalratana N Chutinet A Suwanwela NC. Hyperacute simultaneous cardiocerebral infarction: rescuing the brain or the heart first? Front Neurol 2017;8:664. 29270151 10.3389/fneur.2017.00664 PMC5725403

[3] Tokuda K Shindo S Yamada K. Acute embolic cerebral infarction and coronary artery embolism in a patient with atrial fibrillation caused by similar thrombi. J Stroke Cerebrovasc Dis 2016;25:1797–9. 27105568 10.1016/j.jstrokecerebrovasdis.2016.01.055

[4] Cai XQ Wen J Zhao Y. Acute ischemic stroke following acute myocardial infarction: adding insult to injury. Chin Med J 2017;130:1129–30. 28469112 10.4103/0366-6999.204921 PMC5421187

[5] Karlinski MA Bembenek JP Baranowska A. Noninfectious complications of acute stroke and their impact on hospital mortality in patients admitted to a stroke unit in Warsaw from 1995 to 2015. Neurol Neurochir Pol 2018;52:168–73. 28985991 10.1016/j.pjnns.2017.09.003

[6] Fara MG Stein LK Skliut M. Macrothrombosis and stroke in patients with mild COVID-19 infection. J Thromb Haemost 2020;18:2031–3. 32464707 10.1111/jth.14938 PMC7283879

[7] Levi M Thachil J Iba T. Coagulation abnormalities and thrombosis in patients with COVID-19. Lancet Haematol 2020;7:e438–40. 32407672 10.1016/S2352-3026(20)30145-9 PMC7213964

[8] Driggin E Madhavan MV Bikdeli B. Cardiovascular considerations for patients, health care workers, and health systems during the COVID-19 pandemic. J Am Coll Cardiol 2020;75:2352–71. 32201335 10.1016/j.jacc.2020.03.031 PMC7198856

[9] Middeldorp S Coppens M van Haaps TF. Incidence of venous thromboembolism in hospitalized patients with COVID-19. J Thromb Haemost 2020;18:1995–2002. 32369666 10.1111/jth.14888 PMC7497052

[10] Wang W Xu Y Gao R. Detection of SARS-CoV-2 in different types of clinical specimens. JAMA 2020;323:1843–4. 32159775 10.1001/jama.2020.3786 PMC7066521

[11] Obaid O Smith HR Brancheau D. Simultaneous acute anterior ST-elevation myocardial infarction and acute ischemic stroke of left middle cerebral artery: a case report. Am J Case Rep 2019;20:776–9. 31154453 10.12659/AJCR.916114 PMC6561139

[12] Omar HR Fathy A Rashad R. Concomitant acute right ventricular infarction and ischemic cerebrovascular stroke; possible explanations. Int Arch Med 2010;3:25. 20977759 10.1186/1755-7682-3-25 PMC2974668

[13] Demaerschalk BM Kleindorfer DO Adeoye OM. Scientific rationale for the inclusion and exclusion criteria for intravenous alteplase in acute ischemic stroke: a statement for healthcare professionals from the American Heart Association/American Stroke Association. Stroke 2016;47:581–641. 26696642 10.1161/STR.0000000000000086

[14] Yeo LL Andersson T Yee KW. Synchronous cardiocerebral infarction in the era of endovascular therapy: which to treat first? J Thromb Thrombolysis 2017;44:104–11. 28220330 10.1007/s11239-017-1484-2

[15] Abe S Tanaka K Yamagami H. Simultaneous cardio-cerebral embolization associated with atrial fibrillation: a case report. BMC Neurol 2019;19:152. 31277605 10.1186/s12883-019-1388-1 PMC6612210

[16] Loh E Sutton MS Wun CC. Ventricular dysfunction and the risk of stroke after myocardial infarction. N Engl J Med 1997;336:251–7. 8995087 10.1056/NEJM199701233360403

[17] Arboix A Alióc J. Cardioembolic stroke: clinical features, specific cardiac disorders and prognosis. Curr Cardiol Rev 2010;6:150–61. 21804774 10.2174/157340310791658730 PMC2994107

[18] Nguyen TL Rajaratnam R. Dissecting out the cause: a case of concurrent acute myocardial infarction and stroke. BMJ Case Rep 2011;2011:bcr0220113824. 10.1136/bcr.02.2011.3824 PMC3109691 22693314

[19] Zhou F Yu T Du R. Clinical course and risk factors for mortality of adult inpatients with COVID-19 in Wuhan, China: a retrospective cohort study. Lancet 2020;395:1054–62. 32171076 10.1016/S0140-6736(20)30566-3 PMC7270627

[20] Li Y Wang M Zhou y. Acute cerebrovascular disease following COVID-19: a single center, retrospective, observational study. SSRN Electron J. Available at: 10.2139/ssrn.3550025. PMC7371480 32616524

[21] Klok FA Kruip MJ van der Meer NJ. Incidence of thrombotic complications in critically ill ICU patients with COVID-19. Thromb Res 2020;191:145–7. 32291094 10.1016/j.thromres.2020.04.013 PMC7146714

[22] Boukhris M Hillani A Moroni F. Cardiovascular implications of the COVID-19 pandemic: a global perspective. Can J Cardiol 2020;36:1068–80. 32425328 10.1016/j.cjca.2020.05.018 PMC7229739

[23] Tunç A Ünlübaş Y Alemdar M. Coexistence of COVID-19 and acute ischemic stroke report of four cases. J Clin Neurosci 2020;77:227–9. 32409210 10.1016/j.jocn.2020.05.018 PMC7200342

[24] Mao L Jin H Wang M. Neurologic manifestations of hospitalized patients with coronavirus disease 2019 in Wuhan, China. JAMA Neurol 2020;77:683–90. 32275288 10.1001/jamaneurol.2020.1127 PMC7149362

[25] Huang YH Jiang D Huang JT. SARS-CoV-2 detected in cerebrospinal fluid by PCR in a case of COVID-19 encephalitis. Brain Behav Immun 2020;87:149. 32387508 10.1016/j.bbi.2020.05.012 PMC7202824

[26] Al Saiegh F Ghosh R Leibold A. Status of SARS-CoV-2 in cerebrospinal fluid of patients with COVID-19 and stroke. J Neurol Neurosurg Psychiatry 2020;91:846–8. 32354770 10.1136/jnnp-2020-323522

[27] Zhou Z Kang H Li S. Understanding the neurotropic characteristics of SARS-CoV-2: From neurological manifestations of COVID-19 to potential neurotropic mechanisms. J Neurol 2020;267:2179–84. 32458193 10.1007/s00415-020-09929-7 PMC7249973

[28] Guo Y Korteweg C McNutt MA. Pathogenetic mechanisms of severe acute respiratory syndrome. Virus Res 2008;133:4–12. 17825937 10.1016/j.virusres.2007.01.022 PMC7114157

[29] Baig AM Khaleeq A Ali U. Evidence of the COVID-19 virus targeting the CNS: tissue distribution, host-virus interaction, and proposed neurotropic mechanisms. ACS Chem Neurosci 2020;11:995–8. 32167747 10.1021/acschemneuro.0c00122

[30] Lawrence T. The nuclear factor NF-kappaB pathway in inflammation. Cold Spring Harb Perspect Biol 2009;1:a001651. 20457564 10.1101/cshperspect.a001651 PMC2882124

[31] Holgate ST. Cytokine and anti-cytokine therapy for the treatment of asthma and allergic disease. Cytokine 2004;28:152–7. 15588688 10.1016/j.cyto.2004.07.010

[32] Chung KF. Cytokines as targets in chronic obstructive pulmonary disease. Current Drug Targets 2006;7:675–81. 16787167 10.2174/138945006777435263

[33] Hess DC Eldahshan W Rutkowski E. COVID-19-related stroke. Transl Stroke Res 2020;11:322–5. 32378030 10.1007/s12975-020-00818-9 PMC7202903

[34] Xie X Chen J Wang X. Age- and gender-related difference of ACE2 expression in rat lung. Life Sci 2006;78:2166–71. 16303146 10.1016/j.lfs.2005.09.038 PMC7094566

[35] Chen J Xiao X Chen S. Angiotensin-converting enzyme 2 priming enhances the function of endothelial progenitor cells and their therapeutic efficacy. Hypertension 2013;61:681–9. 23266545 10.1161/HYPERTENSIONAHA.111.00202 PMC4011714

[36] Chen J Zhao Y Chen S. Neuronal over-expression of ACE2 protects brain from ischemia-induced damage. Neuropharmacology 2014;79:550–8. 24440367 10.1016/j.neuropharm.2014.01.004 PMC3992949

[37] Colafrancesco S Alessandri C Conti F. COVID-19 gone bad: a new character in the spectrum of the hyperferritinemic syndrome? Autoimmun Rev 2020;19:102573. 32387470 10.1016/j.autrev.2020.102573 PMC7199723

[38] Yazdanpanah F Hamblin MR Rezaei N. The immune system and COVID-19: Friend or foe? Life Sci 2020;256:117900. 32502542 10.1016/j.lfs.2020.117900 PMC7266583

[39] Tisoncik JR Korth MJ Simmons CP. Into the eye of the cytokine storm. Microbiol Mol Biol Rev 2012;76:16–32. 22390970 10.1128/MMBR.05015-11 PMC3294426

[40] Zhang C Wu Z Li JW. Cytokine release syndrome in severe COVID-19: Interleukin-6 receptor antagonist tocilizumab may be the key to reduce mortality. Int J Antimicrob Agents 2020;55:105954. 32234467 10.1016/j.ijantimicag.2020.105954 PMC7118634

[41] CDC Covid-Response Team. Severe outcomes among patients with coronavirus disease 2019 (COVID-19)—United States, February 12--March 16, 2020. MMWR Morb Mortal Wkly Rep 2020;69:343–6. 32214079 10.15585/mmwr.mm6912e2 PMC7725513

[42] Onder G Rezza G Brusaferro S. Case-fatality rate and characteristics of patients dying in relation to COVID-19 in Italy. JAMA 2020;323:1775–6. 32203977 10.1001/jama.2020.4683

[43] Huang C Wang Y Li X. Clinical features of patients infected with 2019 novel coronavirus in Wuhan, China. Lancet 2020;395:497–506. 31986264 10.1016/S0140-6736(20)30183-5 PMC7159299

[44] Imai Y Kuba K Neely GG. Identification of oxidative stress and toll-like receptor 4 signaling as a key pathway of acute lung injury. Cell 2008;133:235–49. 18423196 10.1016/j.cell.2008.02.043 PMC7112336

[45] Komsiiska D. Oxidative stress and stroke: a review of upstream and downstream antioxidant therapeutic options. Comp Clin Path 2019;28:915–26.

[46] Pokutta S Herrenknecht K Kemler R. Conformational changes of the recombinant extracellular domain of E-cadherin upon calcium binding. Eur J Biochem 1994;223:1019–26. 8055942 10.1111/j.1432-1033.1994.tb19080.x

[47] Yu BP. Cellular defenses against damage from reactive oxygen species. Physiol Rev 1995;74:139–62. 10.1152/physrev.1994.74.1.139 8295932

[48] Crack PJ Taylor JM. Reactive oxygen species and the modulation of stroke. Free Radic Biol Med 2005;38:1433–44. 15890617 10.1016/j.freeradbiomed.2005.01.019

[49] Chan PH Chen S Imaizumi S. New insights into the role of oxygen radicals in cerebral ischemia. Neurochemical Correlates of Cerebral Ischemia 1992.

[50] Albensi BC. What is nuclear factor kappa B (NF-(B) doing in and to the mitochondrion? Front Cell Dev Biol 2019;7:154. 31448275 10.3389/fcell.2019.00154 PMC6692429

[51] Rodrigues Prestes TR Rocha NP Miranda AS. The anti-inflammatory potential of ACE2/angiotensin-(1-7)/mas receptor axis: evidence from basic and clinical research. Curr Drug Targets 2017;18:1301–13. 27469342 10.2174/1389450117666160727142401

[52] South AM Brady TM Flynn JT. ACE2, COVID-19, and ACE inhibitor and ARB use during the pandemic: the pediatric perspective. Hypertension 2020; 10.1161/HYPERTENSIONAHA.120.15291 PMC7289676 32367746

[53] Wilk AJ Rustagi A Zhao NQ. A single-cell atlas of the peripheral immune response to severe COVID-19. medRxiv 2020; 10.1101/2020.04.17.20069930 PMC7382903 32514174

[54] Powers WJ Rabinstein AA Ackerson T. 2018 Guidelines for the early management of patients with acute ischemic stroke: A guideline for healthcare professionals from the American Heart Association/American Stroke Association. Stroke 2018;49:e46–110. 29367334 10.1161/STR.0000000000000158

[55] Akinseye OA Shahreyar M Heckle MR. Simultaneous acute cardio-cerebral infarction: is there a consensus for management? Ann Transl Med 2018;6:7. 29404353 10.21037/atm.2017.11.06 PMC5787723

